# Thoracic Endovascular Aortic Repair (TEVAR) First in Patients with Lower Limb Ischemia in Complicated Type B Aortic Dissection: Clinical Outcome and Morphology

**DOI:** 10.3390/jcm11144154

**Published:** 2022-07-17

**Authors:** Katrin Meisenbacher, Matthias Hagedorn, Denis Skrypnik, Samuel Kilian, Dittmar Böckler, Moritz S. Bischoff, Andreas S. Peters

**Affiliations:** 1 Department of Vascular and Endovascular Surgery, University Hospital Heidelberg, 69120 Heidelberg, Germany; matthias.hagedorn@med.uni-heidelberg.de (M.H.); denis.skrypnik@med.uni-heidelberg.de (D.S.); dittmar.boeckler@med.uni-heidelberg.de (D.B.); moritz.bischoff@med.uni-heidelberg.de (M.S.B.); andreas.peters1@med.uni-heidelberg.de (A.S.P.); 2 Institute of Medical Biometry, University of Heidelberg, 69120 Heidelberg, Germany; kilian@imbi.uni-heidelberg.de

**Keywords:** aortic dissection, thoracic endovascular repair, TEVAR, lower limb ischemia, extremity malperfusion, complicated type B dissection, malperfusion

## Abstract

Acute Type B aortic dissection (TBAD) can cause organ malperfusion, e.g., lower limb ischemia (LLI). Thoracic endovascular aortic repair (TEVAR) represents the standard treatment for complicated TBAD; however, with respect to LLI, data is scant. The aim of this study was to investigate clinical and morphological outcomes in patients with complicated TBAD and LLI managed with a “TEVAR-first” policy. Between March 1997 and December 2021, 731 TEVAR-procedures were performed, including 106 TBAD-cases. Cases with TBAD + LLI were included in this retrospective analysis. Study endpoints were morphological/clinical success of TEVAR, regarding aortic and extremity-related outcome, including extremity-related adjunct procedures (erAP) during a median FU of 28.68 months. A total of 20/106 TBAD-cases (18.8%, 32–82 years, 7 women) presented with acute LLI (12/20 Rutherford class IIb/III). In 15/20 cases, true lumen-collapse (TLC) was present below the aortic bifurcation. In 16/20 cases, TEVAR alone resolved LLI. In the remaining four cases, erAP was necessary. A morphological analysis showed a relation between lower starting point and lesser extent of TLC and TEVAR success. No extremity-related reinterventions and only one major amputation was needed. The data strongly suggest that aTEVAR-first-strategy for treating TBAD with LLI is reasonable. Morphological parameters might be of importance to anticipate the failure of TEVAR alone.

## 1. Introduction

The standard treatment strategy for Stanford Type B aortic dissection (TBAD) consists of the best possible drug therapy, with the aim of adequate blood pressure control [1,2]. However, the disease can take a complicated course: false lumen rupture or organ malperfusion may occur, both requiring emergency treatment, to avoid a fatal outcome. The advances in endovascular therapy in recent decades have led to thoracic endovascular aortic repair (TEVAR) becoming the treatment of choice for complicated TBAD (cTBAD), with lower rates of morbidity and mortality than open repair [1,2,3,4]. With respect to malperfusion, the visceral segment of the aorta can be involved, leading to ischemia of the liver, the small intestine, and the kidneys. Lower limb ischemia (LLI) may appear as an additional or isolated clinical sign. Data from the International Registry of Aortic Dissection (IRAD) show that LLI occurs in 7.1% of patients and represents a risk factor for mortality [5]. From a pathophysiological point of view, malperfusion is caused either by a proximally located aortic true lumen collapse (TLC) or by a dissection of the respective organ-supplying vessel itself. The basic principle of the TEVAR procedure for cTBAD is coverage of the proximal entry tear, to redirect the blood flow into the true lumen, hence overcoming the collapse. Data on the treatment and outcome of cTBAD, in terms of LLI, are sparse. Whether primary TEVAR represents the treatment of choice for LLI in cTBAD is an unresolved question. The study presented here comprises a clinical and morphological analysis of the outcome in patients with cTBAD and LLI managed with a “TEVAR-first” policy.

## 2. Materials and Methods

### 2.1. Study Design

An observational retrospective single-center study was designed to evaluate the impact of TEVAR on LLI in patients with acute cTBAD. This approach includes characterization of both morphological alterations and clinical outcomes. The patients were identified from a departmental prospective database of cases involving any TEVAR procedure, which was approved by the local ethics committee (protocol no. S-158/2015).

### 2.2. Study Cohort

The inclusion criteria comprised cTBAD with clinical and/or radiological signs of LLI and treatment with TEVAR at the authors’ institution between March 1997 and December 2021. Other pathologies, cTBAD with no clinical and/or radiological signs of LLI, and TBAD treated conservatively were excluded, as were patients with missing preoperative and/or postoperative imaging results.

### 2.3. Procedural Data

Up to September 2010, an Axiom-U imaging system (Siemens, Healthineers, Erlangen, Germany) was used; from October 2010 onward, TEVAR took place in a hybrid operating room (Artis zeego multiaxis imaging system; Siemens Healthineers, Erlangen, Germany). From October 2020, the operating theatre was equipped with an Artis pheno angiography system (Siemens Healthineers, Erlangen, Germany). The implantation protocol has been published previously [6,7]. Left subclavian artery (LSA) revascularization was undertaken selectively [8]. The local institutional protocol for the treatment of cTBAD stipulates a TEVAR-first strategy with coverage of the primary entry, irrespective of the clinical presentation and/or associated malperfusion. This comprises the implantation of a single TEVAR device covering the main entry. Hereafter, any further distal extension of TEVAR (usually down to the level of the celiac trunk) or adjunct procedure is based on the findings of subsequent downstream angiography. In general, all TEVAR procedures are performed by senior physicians with extensive TEVAR experience.

### 2.4. Study Endpoints and Definitions

The underlying hypothesis of this study was that TEVAR alone is able to re-establish arterial inflow in patients affected by LLI, without any additional procedures. Therefore, the study endpoints were the morphological and clinical success of TEVAR, with respect to aortic and extremity-related outcome. This approach includes the detailed evaluation of all extremity-related adjunct procedures (erAP) and/or reinterventions, particularly focusing on a detailed morphological characterization, with regard to any association with the technical approach and procedural success.

erAP were defined as any endovascular, open, or hybrid vascular procedure adjunctive to TEVAR with the aim of restoration of blood flow into the affected limb during the index procedure, meeting the definition of the Society for Vascular Surgery and Society of Thoracic Surgeons reporting standards for type B aortic dissections [9].

Extremity-related reinterventions (erRI) were defined as any secondary revascularization-procedure performed for the affected limb after the index procedure (e.g., secondary bypass). 

Extremity-related outcomes were defined as freedom from lower limb amputation and freedom from functional limb impairment. 

**Figure 1:**
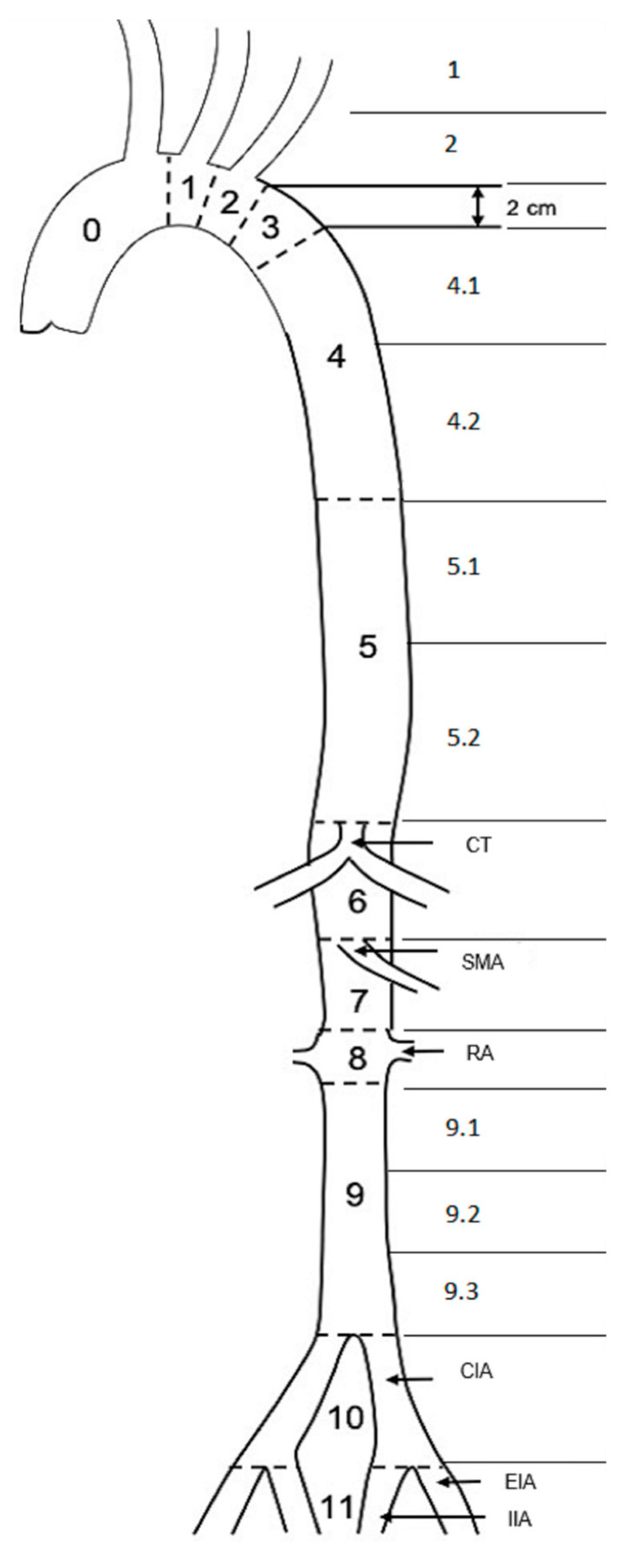
Extended aortic zones; based on Fillinger et al. [12]. CT: celiac trunk; SMA: superior mesenteric artery; RA: renal artery; CIA: common iliac artery; EIA: external iliac artery; IIA: internal iliac artery.

Based on the criteria of White et al., lower extremity malperfusion was defined as missing femoral artery pulses, together with one or more of the following findings: radiographic malperfusion of the affected limb, loss of sensorimotor function, paleness/discoloration of the affected limb, or pain in the affected limb [10]. The Rutherford acute limb ischemia classification system (class I–III) was used for clinical categorization of lower extremity malperfusion [11]. For morphological analysis, the classification of aortic zones introduced by Fillinger et al. [12] was extended to provide a more detailed description of zones 4, 5, and 9 (Figure 1).

### 2.5. Imaging Data and Follow-Up

The institutional protocol includes multidetector electrocardiography-gated computed tomography angiography (CTA) of the entire aorta (supra-aortic branches to femoral arteries) with a 1-mm slice thickness acquired at 60% of the R–R interval, corresponding to late diastole. CTA was carried out before TEVAR; before discharge; at 3 months, 6 months, and 12 months after the index procedure; and annually thereafter [13]. For the data presented here, preoperative and postoperative CTA images were analyzed using certified three-dimensional reconstruction software and centerline measurements (OsiriX PRO; aycan Medical Systems, Rochester, NY, USA). 

Morphological assessment was performed by visualization in a case-by-case fashion, displaying the extent of the dissected segments, the localization of the main entry and the re-entries, and the extent of the TLC, in terms of the aortic zones before the index procedure, after TEVAR (based on the intraoperative angiography), and after treatment (including a potential erAP). In order to visualize any potential distribution pattern in morphology, the cases were secondarily regrouped, comparing the cases treated by TEVAR alone with the TEVAR + erAP cases. Assessment was performed by at least two experienced readers, blinded to all clinical information. In the event of discrepancies, the investigators reached a consensus. In addition, the intraoperative angiography findings following primary TEVAR were evaluated. 

The CTA before TEVAR and at least one postoperative CTA were available in all cases (imaging follow-up [FU] 100%). FU was completed up to January 2022 for all 20 cases (100%). No patient was lost to FU. 

### 2.6. Statistical Analysis

Patient and disease characteristics are described as absolute and relative frequencies for categorical variables and median (range) for continuous data [14]. FU was given by the median, including the 25% and the 75% quantiles (Q1–Q3). Morphological presentation was visualized at patient level and aggregated using further visualization methods. 

## 3. Results

### 3.1. Demographics

**Figure 2:**
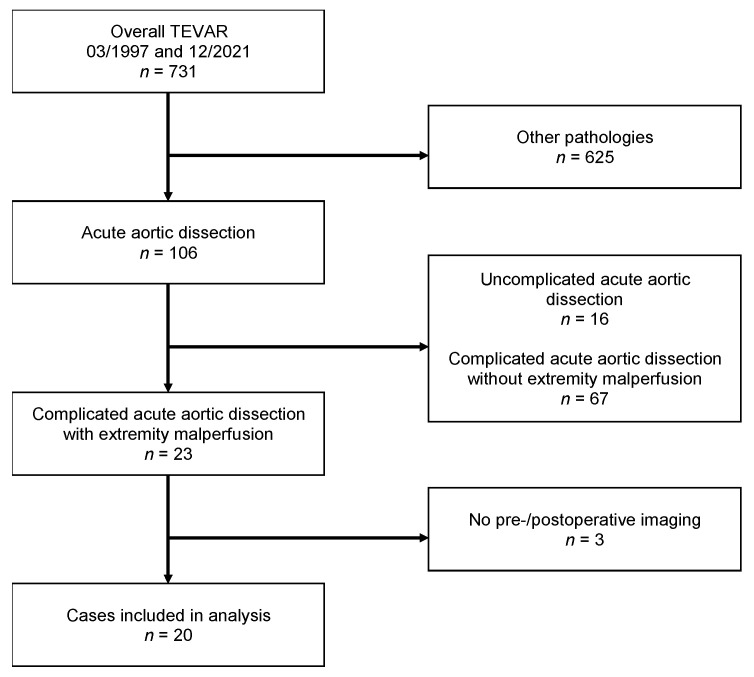
Flowchart of patient selection.

Between March 1997 and December 2021, a total of 731 TEVAR procedures were performed at the authors’ institution, in patients with various aortic pathologies, including 106 cases of cTBAD. Of these 106 patients, 23 (21.7%) presented with LLI. Three cases without available preoperative and/or postoperative imaging results were excluded, leaving 20 patients (20/106; 18.8%; 7 female, 13 male) with a median age of 53 years (range 32–82 years) for analysis (Figure 2). Concomitant visceral/renal malperfusion was found in 80% of cases (16/20), with renal involvement alone in *n* = 7, visceral malperfusion in *n* = 2, and both renal and visceral malperfusion in *n* = 7. The most common comorbidity was arterial hypertension (in 80% of cases, 16/20), while none of the patients had a documented history of peripheral artery disease. Four patients had a history of kidney failure (20%), with a pre-existing need for hemodialysis in *n* = 1 patient. The patient demographics are displayed in Table 1.

### 3.2. Clinical Presentation of LLI

Sixty percent (12/20) of the patients presented with immediately threatened extremities (Rutherford class IIb: *n* = 7, class III: *n* = 5). Eight patients (20%) presented mild symptoms of LLI (Rutherford class IIa). Unilateral LLI was present in 70% (*n* = 14) and bilateral LLI in 30% of cases (*n* = 6). Unilateral malperfusion was seen more frequently in the right (9/14; 64.3%) than in the left limb (5/14; 35.7%). The median creatine kinase (CK) level on admission was 149 U/L (range 52–1231; reference < 190 U/L), and the median serum creatinine (Crea) level was 0.99 mg/dL (range 0.56–5; reference 0.6–1.3 mg/dL).

### 3.3. Procedural Data with Respect to TEVAR and Extremity-Related Adjunct Procedures

All patients except one were treated by TEVAR first, including main entry coverage as an emergency procedure. The proximal landing zone (PLZ) was aortic zone 2 in 16/20 (80%) of the cases; in six cases revascularization of the LSA was performed simultaneously. In one patient, iliac stenting of the affected limb was performed 11 days before TEVAR. Resolution of LLI was achieved immediately in 16/20 (80%) patients after TEVAR, alone with a median of one (range 1–3) implanted device and a median treatment length of 200 mm (range 145–300 mm). 

In four cases (20%), TEVAR failed to restore the blood flow into the affected limb(s). In these cases, additional erAP were performed during the index procedure. A femorofemoral crossover bypass was performed in *n* = 2, iliac stenting in *n* = 1, and infrarenal aortic bare stent implantation in *n* = 1. All procedural details are shown in Table 2. All erAP are displayed case by case in Table 3. 

### 3.4. Extremity-Related Outcomes and Reinterventions 

Fasciotomy was performed in 25% of cases (5/20), of which only one procedure was prophylactic. All fasciotomies were performed during the index procedure. 

While LLI-associated clinical impairment disappeared in 80% of cases (16/20; *n* = 15: TEVAR alone, *n* = 1: TEVAR + erAP), in the remaining four patients (*n* = 1: TEVAR alone, *n* = 3: TEVAR + erAP) postischemic sensorimotor deficits persisted, despite successful reperfusion of the leg. One TEVAR-alone patient (initially presenting with prolonged ischemia of the left leg, Rutherford class 2b) showed peripheral peroneal nerve palsy. In the remaining three cases (initially presenting with Rutherford class 3 and concomitant visceral/renal malperfusion) irreversible ischemia of the affected leg remained, leading in one case to major amputation of the left leg 7 days after the index procedure. The other two patients died of multiorgan failure due to their visceral/renal malperfusion, immediately and 3 days after the index procedure, respectively. 

There was no need for any further extremity-related reintervention during the early or late phase of FU in any case.

### 3.5. Mortality

During a median FU of 28.68 months (Q1 0.6 months, Q3 59.3 months, IQR 58.7 months), six of the 20 patients died (overall mortality 30%). All of them died during the primary hospital stay (in-hospital mortality 30%), an average of 4.5 days after TEVAR (range 0–168 days). Of these six patients, *n* = 3 were treated by TEVAR alone, yielding a mortality rate of 18.75% (3/16) in this group, while the other three were treated with TEVAR and erAP (3/4, 75%). The reasons for death were multiorgan failure in *n* = 4, cardiorespiratory failure in *n* = 1, and liver failure in *n* = 1. None of the patients died due to LLI alone, but rather due to a complicated course of cTBAD with multiorgan involvement.

### 3.6. Morphological Analysis 

The primary entry was located in aortic zone 2 or 3 in 80% of cases (16/20), while only four cases showed a primary entry in zone 4 or 5. The median length of the primary entry was 15 mm (range 6–100 mm). In all cases, multiple re-entries were present (median 3, range 1–9). In *n* = 15/20 (75%) TLC was present below the aortic bifurcation, extending into the affected leg. In 5/20 (15%), the TLC did not reach further than the aortic bifurcation. Post-TEVAR control angiography during the index procedure showed resolution of the TLC in all but four cases (16/20, 80%). In these four cases, LLI persisted in the affected limb/s, so erAP was performed.

**Figure 3:**
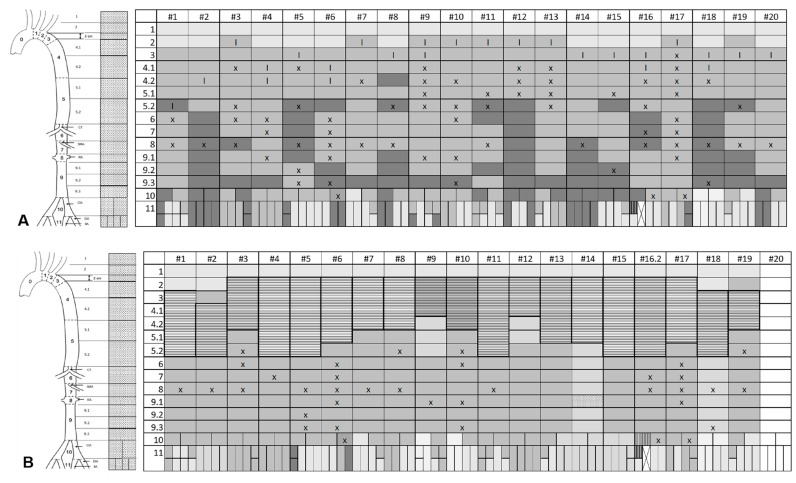

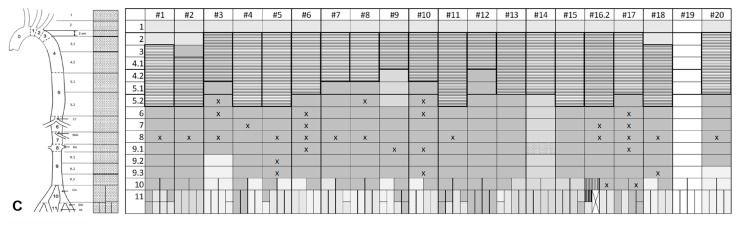
Morphological presentation before TEVAR (**A**), after TEVAR with respect to intraoperative angiography (**B**) and after treatment, including a potential extremity related adjunct procedure (**C**).

**Figure 4:**
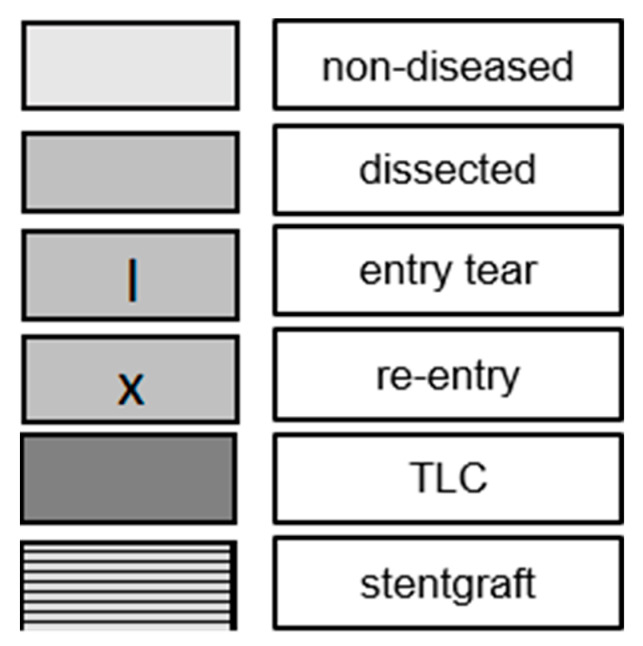
Explanatory caption of Figure 3A–C as well as Figure 5.

**Figure 5:**
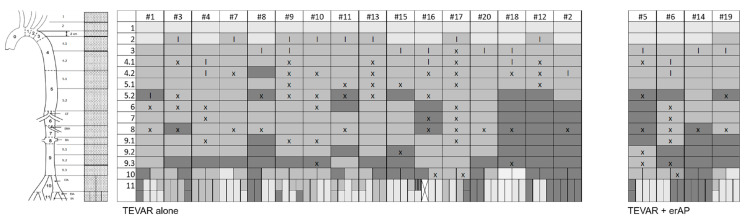
Secondary rearrangement according to morphological distribution patterns, comparing TEVAR alone with TEVAR + extremity related adjunct procedure (erAP) cases.

The morphological presentation is detailed in Table 4 and visualized in case-by-case fashion in Figure 3, which shows the extent of the dissected segments, the localization of the main entry and the re-entries, and the extent of the TLC, in terms of aortic zones before the index procedure (Figure 3A), after TEVAR (based on the intraoperative angiography) (Figure 3B), and after treatment (including a potential erAP) (Figure 3C). The explanatory caption for Figure 3A–C is displayed in Figure 4. Figure 5 shows the initial morphological pattern, comparing the cases subsequently treated by TEVAR alone with the TEVAR + erAP cases. 

**Figure 6:**
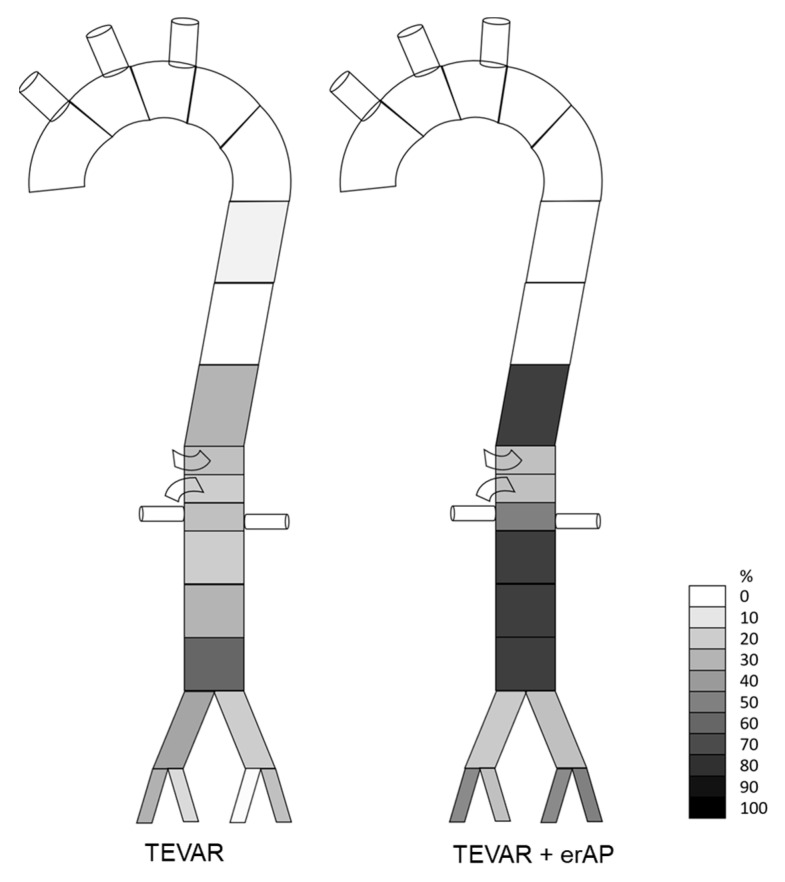
Cumulative frequencies of true lumen collapse, comparing cases treated with TEVAR alone with TEVAR + erAP cases.

**Figure 7:**
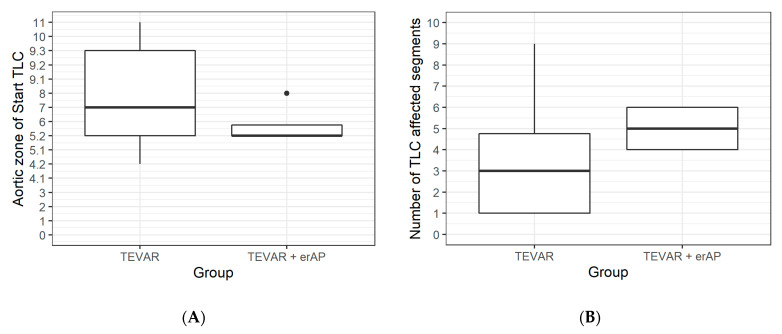
Boxplots representing the starting point (**A**) and the extent of true lumen collapse (TLC) (**B**) in cases treated with TEVAR alone versus TEVAR + erAP cases.

Graphical analysis of the evaluated parameters showed a relation between the starting point and the extent of the TLC regarding TEVAR success. (Figure 6 and Figure 7A,B). 

In addition, the first re-entry was located slightly further downstream in cases treated with TEVAR and erAP. The number of re-entries did not differ between TEVAR alone and TEVAR + erAP. Furthermore, neither the extent of the primary entry tear nor the distribution pattern of the first entry, in terms of aortic zones, showed any apparent difference.

## 4. Discussion

This analysis shows that LLI in cTBAD can be favorably managed with TEVAR alone. In the majority of cases, proximal aortic repair led to a restoration of blood flow into the affected limb, independently of the initial morphological extent of the cTBAD. In 20% (4/20) of the cases presented here, however, LLI persisted, leading to erAP. Nevertheless, the amputation rate was low (*n* = 1) and there was no need for extremity-related reintervention. 

Isolated lower extremity malperfusion accounts for 5.7–30% of cTBAD cases [15,16,17], with bilateral clinical ischemia in more than 50% [15]. Our findings are within this range, with an 18.8% incidence of extremity malperfusion, predominantly in the form of unilateral ischemia. 

The incidence of cTBAD presenting with lower extremity malperfusion has to be interpreted with caution, as in most studies downstream malperfusion syndromes are lumped together, with no figures for extremity malperfusion only. This contributes to the fact that malperfusion of the limbs in aortic dissection is frequently associated with visceral/renal malperfusion [15,18,19,20], as was the case in 80% of our cohort. 

By implication, limb ischemia can be considered as a clinical predictor for the severity of the disease, as it indicates a greater extent of dissection [16,18]. 

Our data show an in-hospital mortality of 30%. However, mortality is related to the consequences of visceral/renal malperfusion, rather than the limb ischemia in itself. In line with our findings, data from the IRAD registry show threefold mortality in patients presenting acute limb ischemia secondary to TBAD, with an association with mesenteric infarction (OR = 6.9; 95% CI 2.5–20; *p* < 0.001), emphasizing the severity of cTBAD with LLI [5,16].

Nevertheless, acute limb ischemia per se represents a serious condition, with hospital mortality rates ranging from 10% to 30%, even in recent years, and with an unchanged 1-year mortality of around 40% [21,22]. Not least, patients presenting with LLI have amputation rates of up to 30%, including a high proportion of amputations above the knee [22,23].

Hence, cTBAD patients presenting with lower extremity malperfusion face both dissection-related and extremity-related sequelae. 

While TEVAR is clearly established as the first-line treatment for cTBAD to solve the aortic problem, guidelines are less explicit regarding TEVAR for the treatment of LLI in aortic dissection [24].

Data from type A aortic dissection cohorts indicate that in most cases extremity ischemia will resolve after proximal aortic repair [20,25,26,27]. In a study of 335 cases of type A dissection, including 18.2% with limb ischemia, only 21.6% of the patients presented with unrelieved ischemia after proximal dissection repair [20].

In general, the evidence on the treatment of lower extremity malperfusion in cTBAD is scant. The available data derive mostly from small cohorts, mainly focusing on any peripheral vascular malperfusion. 

Nevertheless, some authors do support a TEVAR-first approach. Ryan and colleagues reported their experience of TEVAR in a cohort of 68 cTBAD cases, showing malperfusion in various vascular beds, 62% of them with LLI. Concomitant iliac artery stenting was performed in 13 patients (21.3%), matching our 20% rate of erAP [28]. Similarly, Liu et al. compared the treatment outcomes of TEVAR alone versus TEVAR with adjunctive procedures in a collective of 86 type B dissections, including 24 patients with lower extremity malperfusion, in order to identify potential risk factors for the necessity of adjunctive procedures. They were able to show an association between the occurrence of LLI and adjunctive procedures (OR 5.2, 95% CI 1.8–17.4, *p* = 0.003), thereby strengthening the hypothesis that limb ischemia is a marker for more extensive dissection. In their analysis, adjunctive procedures were ultimately necessary in about 20% of cases, whereas in 80% of cases, TEVAR alone was sufficient [18]. Recently, Plotkin et al. reported on 42 patients presenting lower extremity malperfusion in aortic dissection, of whom 26 had type B aortic dissection. The authors compared a “limb-first” to a “dissection-first” approach, with respect to resolution of the malperfusion. They found that 28% of the dissection-first and 50% of the limb-first cases required reintervention to resolve their lower extremity malperfusion. They concluded that proximal aortic repair might be more effective in the treatment of lower extremity malperfusion [17]. This agrees with the findings of our study. 

By contrast, some authors support a limb-first approach. Norton and colleagues propose that aortic fenestration and branch vessel stenting alone may suffice to treat dissection-caused malperfusion, even for LLI, and supporting their argument by reference to the risks involved with TEVAR, such as retrograde aortic dissection and paraplegia [29]. A case series by Corfield et al. suggests femorofemoral crossover grafting as a fast and reliable treatment strategy for acute limb ischemia due to type B aortic dissection [30]. Nevertheless, aortic reintervention in the form of TEVAR was necessary in 56% of their cases during FU. Moreover, femorofemoral crossover grafting is not an option in the presence of bilateral LLI. 

Obviously, TEVAR does not guarantee resolution of lower extremity malperfusion. However, a limb-first approach is not supported by previous publications or, although somewhat implicitly, by our own data. The literature clearly shows that lower extremity malperfusion due to aortic dissection is suggestive of associated malperfusion defects in other peripheral vascular beds [20,31]. Thus, a limb-first approach is time consuming, with the potential for fatal delays. Therefore, the therapeutic approach to cTBAD with LLI must be holistic. This goal is achieved by proximal aortic repair, treating the site from which the disease arises.

While most studies concentrate on clinical endpoints, our objective was to distinguish certain morphological constellations arguing for or against a pure TEVAR approach. Theoretically, branch vessel malperfusions can be divided on pathophysiological grounds, into dynamic obstructions and static obstructions. While dynamic obstruction seems to be more prone to depressurization of the false lumen, dissected branch orifices, characterizing static obstruction, might entail further adjunctive procedures [18,28,29,31]. Precise differentiation is often complicated by the fact that imaging methods, e.g., CTA, are mostly static. 

In evaluating dissection-relevant parameters, such as the primary entry tear, the re-entries, and TLC, we were not able to distinguish any clear difference between cases in which TEVAR alone failed and those where it was successful. Perhaps counterintuitively, TLC appears to start at a more proximal level in cases treated with TEVAR alone, yet in cases requiring erAP, TLC affected a greater number of segments in terms of cumulative frequencies (Figure 6). More predictably, a location of the first re-entry further downstream seemed to be associated with the need for erAP following TEVAR. One may hypothesize that the more re-entries are covered by the endograft, the greater the extent to which the increase in true lumen perfusion is induced, thereby alleviating branch vessel malperfusion. 

For now, there is no apparent morphological pattern enabling advance identification of risk factors for the necessity of erAP. However, while the analysis showed that TEVAR is sufficient in the majority of cases, there are several therapeutic options in the event of clinical and/or morphological TEVAR failure, in terms of persisting malperfusion. While the appropriate treatment for complicated aortic dissection continues to depend on individual patient-related and surgeon-related factors, the morphological approach to several parameters presented here may serve as a blueprint for ongoing research.

### Limitations

In addition to the retrospective monocentric character and the inherent restrictions of retrospective data, this study shows several limitations. 

Because data retrieved from a TEVAR database including only patients who have undergone placement of a thoracic stent graft, our cohort included no patients with limb revascularization procedures only; a potential control group. However, due to the long-standing institutional TEVAR-first approach in the treatment of cTBAD, missed cases are unlikely. The indications for and timing of both TEVAR and erAP were at the discretion of the treating surgeons and cannot be reconstructed for individual cases. Due to the long inclusion period, the CTA data may differ in terms of imaging quality. Given the small number of cases, a distinctive statistical analysis was not performed. Despite extensive efforts and detailed assessment, we were not able to identify a consistent morphological pattern. 

Nevertheless, considering the limited nature of the literature regarding detailed morphological assessment to date, this article represents an important addition to the existing data, strengthening the role of TEVAR in cTBAD with LLI.

## 5. Conclusions

The data presented here strongly suggest that TEVAR-first-and-see is an adequate strategy for the treatment of cTBAD presenting with LLI. Morphological parameters could be of importance in terms of risk factors, predicting failure of TEVAR alone. In order to recognize morphological patterns, potentially allowing for prospective individualization of treatment, future large-scale multicenter collection of imaging data is imperative.

## Figures and Tables

**Table 1:** Demographics.

	Total (*n* = 20)
Age, median (median/range; years)	53 (32–82)
Gender (male/female)	13/7
ASA classification (median/range)	3 (1–5)
Heart failure	1 (5%)
Arterial Hypertension	16 (80%)
History of myocardial infarction	2 (10%)
Coronary artery disease	3 (15%)
Carotid artery stenosis	0 (0%)
Peripheral artery occlusive disease	0 (0%)
History of stroke	0 (0%)
COPD	2 (10%)
Diabetes mellitus	1 (5%)
BMI > 30 kg/m^2^	6 (30%)
Renal insufficiency *	4 (20%)
Need for hemodialysis	1 (5%)
History of smoking	5 (25%)
Previous aortic surgery/intervention	2 (10%)
Abdominal aorta	1 (5%)
Thoracic aorta	1 (5%)

Categorical data are *n* (number)/%. * (creatinine > 1.2 mg/dL). BMI: body mass index; COPD: chronic obstructive pulmonary disease.

**Table 2:** Procedural data.

	Total (*n* = 20)	TEVAR Alone (*n* = 16)	TEVAR + erAP (*n* = 4)
Duration of procedure (min; median/range)	162 (83–435)	159 (83–384)	296 (125–435)
Radiation time (min; median/range)	13 (7–38)	13 (7–38)	19 (12–32)
Contrast agent volume (mL; median/range)	220 (90–480)	215 (90–480)	220 (220–250)
Dose area product (uGy/m^2^; median/range)	38,206 (2215–257,955)	40,500 (2215–257,955)	23,685 (8102–80,428)
CSFD	8 (40%)	6 (37.5%)	2 (50%)
Preoperatively	6 (30%)	5 (31.25%)	1 (25%)
Postoperatively	2 (10%)	1 (6.25%)	1 (25%)
Implanted device			
Gore^®^ TAG^®^	2 (10%)	1 (6.25%)	1 (25%)
Gore^®^ CTAG^®^	11 (55%)	10 (62.5%)	1 (25%)
Gore^®^ CTAG^®^ ACS	7 (35%)	5 (31.25%)	2 (50%)
No. of implanted devices (median/range)	1 (1–3)	1 (1–3)	1 (1–1)
1 device	14 (70%)	10 (62.5%)	4 (100%)
2 devices	2 (10%)	2 (12.5%)	0
3 devices	1 (5%)	1 (6.25%)	0
Covered aortic length (mm)	200 (145–300)	200 (145–300)	200 (150–200)
Access			
Transfemoral cutdown	17 (85%)	14 (87.5%)	3 (75%)
Transfemoral + transbrachial	3 (15%)	2 (12.5%)	1 (25%)
LSA-coverage	16 (80%)	12 (75%)	4 (100%)
Primary LSA revascularization	6 (30%)	5 (31.25%)	1 (25%)
AIHA/rapid pacing	1/9	1/8	0/1
Time to TEVAR (d; median/range)	1 (0–11)	1 (0–11)	0 (0–12)
Length of hospital stay (d; median/range)	19.5 (1–98)	23 (2–98)	11 (1–19)
Length of ICU stay (d; median/range)	7 (1–32)	9 (2–32)	3 (1–19)

Categorical data are *n* (number)/%. ACS: active control system; AIHA: adenosine-induced heart arrest; CSFD: cerebrospinal fluid drainage; d: days; ICU: intensive care unit; LSA: left subclavian artery; min: minutes; mm: millimeter.

**Table 3:** Extremity-related adjunct procedures.

#	Affected Limb	TLC Reaching into Affected Iliac Vessel?	Type of erAP	Angiography before erAP?	Covered Aortic Length (mm) /DLZ	FU
19	Right	No	Femorofemoral COBP left to right	No	150/4.1	Death (MOF)
14	Both	Yes	Infrarenal aortic bare stent	Yes	200/5.1	Death (MOF)
6	Left	Yes	1. Stent left CIA/EIA left 2. Femorofemoral COBP right to left	Yes	200/5.1	Death (CRF)
5	Right	Yes	Stent EIA left	Yes	200/5.1	✔

CIA: common iliac artery; COPB: cross-over bypass; CRF: cardiorespiratory failure; d: days; DLZ: distal landing zone; EIA: external iliac artery; erAP: extremity-related adjunct procedure; mm: millimeter; MOF: multi-organ failure; FU: follow-up; ✔: alive in FU.

**Table 4:** Morphological data before TEVAR.

	Total (*n* = 20)	TEVAR Alone (*n* = 16)	TEVAR + erAP (*n* = 4)
Proximal ET			
Zone 2	10 (50%)	8 (50%)	2 (50%)
Zone 3	6 (30%)	5 (31.25%)	1 (25%)
Zone 4	3 (15%)	2 (12.5%)	1 (25%)
Zone 5	1 (5%)	1 (6.25%)	0
Extent of ET (mm; median, range)	15 (6–100)	15 (6–36)	14.2 (12–100)
No. of Re-Entries (median, range)	3 (1–9)	3 (1–9)	3.5 (1–6)
Start TLC/Zone (median, range)	6 (4.2–11)	7 (4.2–11)	5.2 (5.2–8)
No. of TLC affected segment (median, range	4 (1–9)	3 (1–9)	5 (4–6)
One segment TLC	6 (30%)	6 (37.5%)	0
2 + segments TLC	14 (70%)	10 (62.5%)	4 (100%)
Concomitant visceral organ malperfusion	16 (80%)	12 (75%)	4 (100%)
TLC in affected leg	15 (75%)	12 (75%)	3 (75%)

Categorical data are *n* (number)/%. ET: entry tear; mm: millimeter; TLC: true lumen collapse.

## Data Availability

Data are available in the manuscript and on personal request to the corresponding author.

## References

[1] Riambau V., Böckler D., Brunkwall J., Cao P., Chiesa R., Coppi G., Czerny M., Fraedrich G., Haulon S., Jacobs M.J. (2017). Editor’s Choice-Management of Descending Thoracic Aorta Diseases: Clinical Practice Guidelines of the European Society for Vascular Surgery (ESVS). Eur. J. Vasc. Endovasc. Surg..

[2] MacGillivray T.E., Gleason T.G., Patel H.J., Aldea G.S., Bavaria J.E., Beaver T.M., Chen E.P., Czerny M., Estrera A.L., Firestone S. (2022). The Society of Thoracic Surgeons/American Association for Thoracic Surgery Clinical Practice Guidelines on the Management of Type B Aortic Dissection. Ann. Thorac. Surg..

[3] Fattori R., Tsai T.T., Myrmel T., Evangelista A., Cooper J.V., Trimarchi S., Li J., Lovato L., Kische S., Eagle K.A. (2008). Complicated acute type B dissection: Is surgery still the best option?: A report from the International Registry of Acute Aortic Dissection. JACC Cardiovasc. Interv..

[4] Zeeshan A., Woo E.Y., Bavaria J.E., Fairman R.M., Desai N.D., Pochettino A., Szeto W.Y. (2010). Thoracic endovascular aortic repair for acute complicated type B aortic dissection: Superiority relative to conventional open surgical and medical therapy. J. Thorac. Cardiovasc. Surg..

[5] Suzuki T., Mehta R.H., Ince H., Nagai R., Sakomura Y., Weber F., Sumiyoshi T., Bossone E., Trimarchi S., Cooper J.V. (2003). Clinical profiles and outcomes of acute type B aortic dissection in the current era: Lessons from the International Registry of Aortic Dissection (IRAD). Circulation.

[6] Geisbüsch P., Hoffmann S., Kotelis D., Able T., Hyhlik-Dürr A., Böckler D. (2011). Reinterventions during midterm follow-up after endovascular treatment of thoracic aortic disease. J. Vasc. Surg..

[7] Böckler D., Bischoff M.S., Kronsteiner D., Skrypnik D., Meisenbacher K. (2021). Outcome analysis of the Gore Conformable Thoracic Stent Graft with active control system for the treatment of arch and descending thoracic aortic disease. Eur. J. Cardiothorac. Surg..

[8] Kotelis D., Geisbüsch P., Hinz U., Hyhlik-Dürr A., von Tengg-Kobligk H., Allenberg J.R., Böckler D. (2009). Short and midterm results after left subclavian artery coverage during endovascular repair of the thoracic aorta. J. Vasc. Surg..

[9] Lombardi J.V., Hughes G.C., Appoo J.J., Bavaria J.E., Beck A.W., Cambria R.P., Charlton-Ouw K., Eslami M.H., Kim K.M., Leshnower B.G. (2020). Society for Vascular Surgery (SVS) and Society of Thoracic Surgeons (STS) reporting standards for type B aortic dissections. J. Vasc. Surg..

[10] White R.A., Miller D.C., Criado F.J., Dake M.D., Diethrich E.B., Greenberg R.K., Piccolo R.S., Siami F.S. (2011). Report on the results of thoracic endovascular aortic repair for acute, complicated, type B aortic dissection at 30 days and 1 year from a multidisciplinary subcommittee of the Society for Vascular Surgery Outcomes Committee. J. Vasc. Surg..

[11] Rutherford R.B., Baker J., Ernst C., Johnston K., Porter J.M., Ahn S., Jones D.N. (1997). Recommended standards for reports dealing with lower extremity ischemia: Revised version. J. Vasc. Surg..

[12] Fillinger M.F., Greenberg R.K., McKinsey J.F., Chaikof E.L. (2010). Society for Vascular Surgery Ad Hoc Committee on TRS. Reporting standards for thoracic endovascular aortic repair (TEVAR). J. Vasc. Surg..

[13] Meisenbacher K., Böckler D., Geisbüsch P., Hank T., Bischoff M.S. (2020). Preliminary results of spot-stent grafting in Stanford type B aortic dissection and intramural haematoma. Eur. J. Cardiothorac. Surg..

[14] Hickey G.L., Dunning J., Seifert B., Sodeck G., Carr M.J., Burger H.U., Beyersdorf F. (2015). Statistical and data reporting guidelines for the European Journal of Cardio-Thoracic Surgery and the Interactive CardioVascular and Thoracic Surgery. Eur. J. Cardiothorac. Surg..

[15] Gargiulo M., Massoni C.B., Gallitto E., Freyrie A., Trimarchi S., Faggioli G., Stella A. (2014). Lower limb malperfusion in type B aortic dissection: A systematic review. Ann. Cardiothorac. Surg..

[16] Henke P.K., Williams D.M., Upchurch G.R., Proctor M., Cooper J.V., Fang J., Nienaber C.A., Isselbacher E.M., Fattori R., Dasika N. (2006). Acute limb ischemia associated with type B aortic dissection: Clinical relevance and therapy. Surgery.

[17] Plotkin A., Vares-Lum D., Magee G.A., Han S.M., Fleischman F., Rowe V.L. (2021). Management strategy for lower extremity malperfusion due to acute aortic dissection. J. Vasc. Surg..

[18] Liu Y., Jiang X., Chen B., Jiang J., Ma T., Dong Z., Fu W. (2022). Risk factors and treatment outcomes for type B aortic dissection with malperfusion requiring adjunctive procedures after thoracic endovascular aortic repair. J. Vasc. Surg..

[19] Lu W., Fu W., Wang L., Guo D., Xu X., Chen B., Jiang J. (2021). Morphologic characteristics and endovascular management of acute type B dissection patients with superior mesenteric artery involvement. J. Vasc. Surg..

[20] Charlton-Ouw K.M., Sritharan K., Leake S.S., Sandhu H.K., Miller C.C., Azizzadeh A., Safi H.J., Estrera A.L. (2013). Management of limb ischemia in acute proximal aortic dissection. J. Vasc. Surg..

[21] Bjorck M., Earnshaw J.J., Acosta S., Goncalves F.B., Cochennec F., Debus E.S., Hinchliffe R., Jongkind V., Koelemay M.J.W., Menyhei G. (2020). Editor’s Choice-European Society for Vascular Surgery (ESVS) 2020 Clinical Practice Guidelines on the Management of Acute Limb Ischaemia. Eur. J. Cardiothorac. Surg..

[22] Duran M., Oberhuber A., Schelzig H., Simon F. (2016). Aktueller Forschungsstand zur akuten Extremitätenischämie. Gefässchirurgie.

[23] Grip O., Kuoppala M., Acosta S., Wanhainen A., Akeson J., Bjorck M. (2014). Outcome and complications after intra-arterial thrombolysis for lower limb ischaemia with or without continuous heparin infusion. Br. J. Surg..

[24] Erbel R., Aboyans V., Boileau C., Bossone E., Bartolomeo R.D., Eggebrecht H., Evangelista A., Falk V., Frank H., Gaemperli O. (2014). 2014 ESC Guidelines on the diagnosis and treatment of aortic diseases: Document covering acute and chronic aortic diseases of the thoracic and abdominal aorta of the adult. The Task Force for the Diagnosis and Treatment of Aortic Diseases of the European Society of Cardiology (ESC). Eur. Heart J..

[25] Fann J.I., Sarris G.E., Mitchell R.S., Shumway N.E., Stinson E.B., Oyer P.E., Miller D.C. (1990). Treatment of patients with aortic dissection presenting with peripheral vascular complications. Ann. Surg..

[26] Girardi L.N., Krieger K.H., Lee L.Y., Mack C.A., Tortolani A.J., Isom O.W. (2004). Management strategies for type A dissection complicated by peripheral vascular malperfusion. Ann. Thorac. Surg..

[27] Lauterbach S.R., Cambria R.P., Brewster D.C., Gertler J.P., LaMuraglia G.M., Isselbacher E.M., Hilgenberg A.D., Moncure A.C. (2001). Contemporary management of aortic branch compromise resulting from acute aortic dissection. J. Vasc. Surg..

[28] Ryan C., Vargas L., Mastracci T., Srivastava S., Eagleton M., Kelso R., Clair D., Sarac T.P. (2013). Progress in management of malperfusion syndrome from type B dissections. J. Vasc. Surg..

[29] Norton E.L., Williams D.M., Kim K.M., Khaja M.S., Wu X., Patel H.J., Deeb G.M., Yang B. (2020). Management of acute type B aortic dissection with malperfusion via endovascular fenestration/stenting. J. Thorac. Cardiovasc. Surg..

[30] Corfield L., McCormack D.J., Bell R., Taylor P., Reidy J. (2014). Role of the femorofemoral crossover graft in acute lower limb ischemia due to acute type B aortic dissection. Vascular.

[31] Wang G.J., Jackson B.M., Damrauer S.M., Kalapatapu V., Glaser J., Golden M.A., Schneider D. (2021). Unique characteristics of the type B aortic dissection patients with malperfusion in the Vascular Quality Initiative. J. Vasc. Surg..

